# Supplementary material for: Cognitive Remediation in Virtual Environments for Patients with Schizophrenia and Major Depressive Disorder: A Feasibility Study
Source: Int J Environ Res Public Health. 2021 Aug 28;18(17):9081. doi: 10.3390/ijerph18179081 (PMC8431186; doi:10.3390/ijerph18179081)
Supplement: Supplementary file 1 [file ijerph-18-09081-s001.zip › ijerph-1317059-supplementary.pdf]

Supplement 1

Table S1: Psychiatric Diagnosis and Medication

Schizophrenia or other primary psychotic disorder

Fifteen participants were diagnosed with schizophrenia, five participants with acute and transient psychotic disorders, one participant suffered from delusional disorder, one participant was diagnosed with schizoaffective disorder.

One participant was medicated with typical antipsychotics. Nine participants were medicated with a combination of typical and atypical antipsychotics and twelve participants were medicated with atypical antipsychotics.

Depressive disorder

Four participants were diagnosed with a depressive episode and two with recurrent depressive disorder.

Two participants were medicated with SNRI antidepressants, two participants were medicated with a combination of SNRI and SARI antidepressants, and two participants with a combination of SSRI and SARI antidepressants.

Supplement 2

Table S1: Feedback questionnaire

1. Did you enjoy the program on the computer? *(1 - I did not enjoy it at all; 3 - not sure; 5 - I really enjoyed it)*
2. Did you enjoy standard paper-pencil treatment? *(1 - I did not enjoy it at all; 3 - not sure; 5 - I really enjoyed it)*
3. How difficult was the program on the computer for you? *(1 - Very easy; 3 - Neither easy not difficult; 5 - Very difficult)*
4. How difficult was the standard paper-pencil treatment for you? *(1 - Very easy; 3 - Neither easy not difficult; 5 - Very difficult)*
5. How beneficial did the program on the computer seem to you? *(1 - Totally useless; 3 - Neither useless not beneficial;5 - Very beneficial)*
6. How beneficial did the standard paper-pencil treatment seem to you? *(1 - Totally useless; 3 - Neither useless not beneficial;5 - Very beneficial)*
7. I feel that after the program on the computer, I have improved my memory, attention, or speech skills have improved. *(1 - No improvement at all; 3 - Not sure; 5 - Significant improvement)*
8. I feel that after the standard paper-pencil treatment I have improved my memory, attention, or language skills. *(1 - No improvement at all; 3 - Not sure; 5 - Significant improvement)*
9. Do you want to tell us something about the program on the computer? *(open question)*
10. Do you want to tell us something about standard paper-pencil treatment? *(open question)*
11. Did you enjoy shopping in the supermarket? *(1- I did not enjoy it at all; 3 - not sure; 5 - I really enjoyed it)*
12. Did you enjoy collecting items in the house? *(1- I did not enjoy it at all; 3 - not sure; 5 - I really enjoyed it)*
13. Did you enjoy the shooting gallery? *(1- I did not enjoy it at all; 3 - not sure; 5 - I really enjoyed it)*
14. Would you like to repeat the program on the computer in the future? *(1 - I definitely would not want to repeat the training; 3 - Not sure;5 - I would really like to repeat the training)*
15. Would you like to repeat the standard paper-pencil program in the future? *(1 - I definitely would not want to repeat the training; 3 - Not sure;5 - I would really like to repeat the training*)
16. Did you use any strategy or specific procedure when remembering the list from the supermarket? *(yes-no)*
17. What strategy did you use to memorize objects? *(Checkboxes)*
    a. *Memorizing*
    b. *Classifying the items (fruits, vegetables, cosmetics...)*
    c. *Visualizing objects*
    d. *Associating between objects (eg. spoon belong on a plate)*
    e. *Imagining a route or the position of the items in the supermarket*
    f. *Creating a story from objects*
    g. *Another answer (fill in):*
